# Subject—What Is Our Duty to Those Who Enter Our Offices as Students, Relative to Our Professon

**Published:** 1872-06

**Authors:** George A. Mills


### Subject.—What is Our duty to Those who Enter our Offices as Students, Relative to our Professon.

GEORGE A. MILLS.

Every honorable calling is worthy of the faithful guardianship of those who accept it, and if there be those who do not prize it enough to guard its honor, they are most certainly unworthy a place in its circle. Yea! more. They are barnacles that will retard its progress and do prove false lights, leading many astray. Taking this view as a preface to the subject (in which I think all will concur), I will at once put the question: Is the calling of the profession of dentistry an honorable one? It is beyond a doubt as much so as any on this planet.

If I were asked what callings in the necessities of the human family were of the most importance, I should say the physician of the soul and body, in which are involved the most solemn responsibilities of *this* life, and that to come. The dentist—in his fullest sense—is a physician of the body, and in my opinion, because this *truth* is not kept in full view at all times, is one of the greatest causes why we are not esteemed by all above the *tinker*.

To raise our profession in its truest sense to one, we *must* guard its open door. Now I will proceed to answer the question proposed, "What is our duty to those who enter offices as students, relative to our profession?" Our first duty, and perhaps the only one, is at once to seek to impress them with the fact that it is an honorable calling, and if we can be successful in committing to them a true sense of its importance, and its responsibilities, we have placed the entering wedge, which is to open up the way to their professional life. We are all well aware that those who seek an entrance to our profession cannot at a glance see what is before them. Those only who have had the experience can know much of the nature of the requirements of the calling. Before I entered I sought the counsel of one who had a few years of experience. He said to me, "There are many difficulties to be met, but the calling is worthy of your best efforts." In another manner I might reply to the question, by telling you how I meet those who apply to me as students.

I go through a primary examination by questions, viz: Why do you choose the calling? How long do you expect it will take you to be able to render creditable service to those who may apply to you? What is your age? What have been your educational advantages? What have you turned your particular attention to up to this time? Are you in good health? What are your pecuniary resources? Do you expect to give your time while under pupilage, or to be paid? How good a dentist do you aspire to be? Those I would consider sufficient to draw out the applicant, and open the way to counsel. The nature of the answers would reveal much of the *real* interest in the pursuit before him. I believe we have arrived at a period in our speciality when we should more unit edly give this matter our particular attention. The position which the calling of dentistry *must* occupy in the coming years, in order to meet the demands that *will* be made upon it, must of necessity look to the *right directing* of those who are to compose the dental ranks of the future. The course pursued by the large proportion of our profession, relative to the taking of students, would not reflect much credit if all were brought to light. Young men, as a rule, are *hired* to enter our ranks, and are required to make *little* if any sacrifice. If in any other profession there is an application for admission, requirements are demanded, and must be met.

Now by the course pursued by a majority of the dentists, I hold it is a decided detriment to the applicant, as well as the profession, arising from this fact, that the way is made easy; the demands and the great importance, which if not now quite apparent to many, are withheld. Study is one of the vital points to which the mind of the student must be directed, and mental training by *us*, if we are able, if not, by those who *are competent*. When I say study I do not mean simply reading, as it is much the custom of the present time to do *superficially*, but study that will set the mind to thinking. When I say teaching I do not mean that of prosy professors and light titilating of the mind, but that which may be given by him who feels something of the importance of the task committed to him, and only from such is there much that is worthy a place in the store-house of knowledge.

A scriptural maxim, which is fraught with much meaning, is worthy of our notice, "Stand by thyself." The child must creep ere it can walk and that by its own efforts. No one however much it may have the little one at heart, can creep for it. We do not need to go far away from home to find those springing up around us who have literally been carried but a few months in the arms of our indulgent profession, and have been dumped down in their swaddling clothes, and the community acknowledge them as a part of the dental profession. Are they prepared to meet the demands of the hour? I answer most emphatically, No! and it is not their fault wholly. Why? only because they have been sent out, and point to their *alma mater* without any attempt on our part to disinherit them. *This* is an evil, and like all morbid growths will prove a very unwelcome companion to discard from our now unsteady bark if we do not arouse ourselves to the position from which we can clearly see the right course to pursue. I do not think the majority wrongly disposed in this matter. Yea! more, I think many are under conviction that I am not overstating the condition we are in. If you admit the truth of my statements, then I say you must, to be at all consistent, *act*, and that speedily and wisely. I have no rule by which we might act, to present at this time. In stating these thoughts not (perhaps) very concisely arranged, I have an earnestness of spirit, which, could they find in you a companionship, they would become fruithful. What students need is the *naked truth.* If this is presented to them, then they start on a right basis, and all dogmas will find little encouragement in their minds. We need truth more than we need legislation. I do not think so much of that method for the purging of the profession. My opinion is, that would we put the same amount of energy in the direction which I have written, it would be more beneficial. I do not like arbitrary rule.

I trust this subject will receive the attention it demands ere long. With the light I have coming to me from day to day, my labor can only be in the direction that will teach mankind the blessings of obedience to a law from within. Then realizing this truth, *for it is the biggest one extant*, real success can come only by holding up this line of teaching to those who seek an entrance to our calling, according to the light we individually have. I am aware that what I have said is but a spark toward the final illumination. But we have had in the conflagration of Chicago the fulfillment of those words, "behold how great a matter a little fire kindleth." Then if you shall use what I have said wisely, it can be made to prove that the ruins of Chicago are not in vain. There is another vital point that must not be overlooked in our direction of the dental student. It is this. Be cautious that we do not, by word or deed, be-little our calling. The longer my observation extends, I am more convinced that we little know what great counter parts we are to become as the exponents of scientific truth.

(What Henry Ward Beecher proclaimed but a short time since in his pulpit, in referring to the teachings of Darwin and Huxley, saying, that scientific men as yet little comprehend the mission they are to work out for the benefit of the human race), is without a doubt the truth. I accept it as such; and it is high time that each one of us stripped ourselves of all dogmatic and hereditary teachings, which hinder us from the acceptance of that which will nourish us in mind and spirit. For if we do not grow in this direction, we do not in anything that is elevating.

So I take it, I do not disagree with any class of thinkers, who would declare, if asked what is the meaning of the redemption of the human race? would answer from all quarters, *elevation.* Now what does it behoove us to raise?—a high or low standard? Some say that our calling does not demand so much effort as some seem to put forth. Let us see. What have we in hand? For what is dental histology, physiology, and pathology? What will they lead us to undersrand when their mission is fully revealed! Have we not the men in our ranks who bid fair to rank with those of any profession? If they do not already, do you think they will bring their *mite* to the general gathering in of all contributions, which is one day to make up and solve the now momenttous problem, the law of *organization.* I believe, and I will say more, I *know,* from what comes welling up within me, that with the assistance of *divine* inspiration, we are to have demonstrated to us yet in this life, that the law of organization and the law of spirit must be co-equal, and only tlll then shall we be able to marry our *intellects* and our affections, and stand out perfected, as it was the design of the Creator in the beginning.

Now I think you will admit that I have, in my *own opinion,* raised a high point of attainment for our now quite obscure profession, to attain to co-workers in the redemption of the human soul. If we accept it, *our* duty to students will be fully apparent.

